# Conservation and Diversification of Circadian Rhythmicity Between a Model Crassulacean Acid Metabolism Plant *Kalanchoë fedtschenkoi* and a Model C_3_ Photosynthesis Plant *Arabidopsis thaliana*

**DOI:** 10.3389/fpls.2018.01757

**Published:** 2018-11-28

**Authors:** Robert C. Moseley, Ritesh Mewalal, Francis Motta, Gerald A. Tuskan, Steve Haase, Xiaohan Yang

**Affiliations:** ^1^Department of Biology, Duke University, Durham, NC, United States; ^2^Department of Forest Ecosystems and Society, Oregon State University, Corvallis, OR, United States; ^3^Department of Mathematical Sciences, Florida Atlantic University, Boca Raton, FL, United States; ^4^Biosciences Division, Oak Ridge National Laboratory, Oak Ridge, TN, United States; ^5^DOE Center for Bioenergy Innovation, Oak Ridge National Laboratory, Oak Ridge, TN, United States; ^6^The Bredesen Center for Interdisciplinary Research and Graduate Education, University of Tennessee, Knoxville, Knoxville, TN, United States

**Keywords:** Arabidopsis, crassulacean acid metabolism, circadian clock, *Kalanchoë fedtschenkoi*, orthologs, phase shifts, photosynthesis, rhythmicity

## Abstract

Crassulacean acid metabolism (CAM) improves photosynthetic efficiency under limited water availability relative to C_3_ photosynthesis. It is widely accepted that CAM plants have evolved from C_3_ plants and it is hypothesized that CAM is under the control of the internal circadian clock. However, the role that the circadian clock plays in the evolution of CAM is not well understood. To identify the molecular basis of circadian control over CAM evolution, rhythmic gene sets were identified in a CAM model plant species (*Kalanchoë fedtschenkoi*) and a C_3_ model plant species (*Arabidopsis thaliana*) through analysis of diel time-course gene expression data using multiple periodicity detection algorithms. Based on protein sequences, ortholog groups were constructed containing genes from each of these two species. The ortholog groups were categorized into five gene sets based on conservation and diversification of rhythmic gene expression. Interestingly, minimal functional overlap was observed when comparing the rhythmic gene sets of each species. Specifcally, metabolic processes were enriched in the gene set under circadian control in *K. fedtschenkoi* and numerous genes were found to have retained or gained rhythmic expression in *K. fedtsechenkoi*. Additonally, several rhythmic orthologs, including CAM-related orthologs, displayed phase shifts between species. Results of this analysis point to several mechanisms by which the circadian clock plays a role in the evolution of CAM. These genes provide a set of testable hypotheses for future experiments.

## Introduction

All organisms confront environmental fluctuations on a daily basis, including changes in light, temperature, predation risk and water availability. Many of these changes exhibit predictable diurnal oscillations, which incentivized organisms to evolve internal timekeeping systems in anticipation of environmental changes. As a result, an emergent circadian clock enabled organisms to synchronize their biological activity with external cues (Bell-Pederson et al., 2005). The circadian clock is important for plant survival, due to their sessile habit, and controls time-of-day specific biology. Greenham and McClung (2015) demonstrated that defense pathways become subordinate to the circadian clock, allowing the plant to activate defense pathways to the time of day when the threat posed by herbivores is maximal. Numerous processes have been documented to be under the control of the circadian clock; however, the extent to which the clock controls basic physiology is still not well understood (Piechulla, 1988; Merida et al., 1999; Thain et al., 2000; Hotta et al., 2007; Mallona et al., 2011; McClung, 2013; Ming et al., 2015).

Crassulacean acid metabolism (CAM) provides a unique physiological system for the study of circadian control over physiology due to a temporal separation of CO_2_ fixation and inverted day/night stomatal movement patterns. It is widely accepted that CAM photosynthesis plants have evolved from C_3_ photosynthesis plants (Yang et al., 2015, 2017; Yin et al., 2018). CAM photosynthesis results in improved photosynthetic and water-use efficiency, relative to C_3_ photosynthesis, and it is hypothesized that the strict temporal control of CAM processes is maintained by the circadian clock in CAM plants (Wyka et al., 2004; Boxall et al., 2005; Hartwell, 2005, 2006; von Caemmerer and Griffiths, 2009; Ming et al., 2015). Evidence from several levels of biology have been reported supporting this hypothesis. At the physiological level, Wilkins (1992) demonstrated that stomatal conductance in CAM plants displays robust 24 h rhythms in constant light and temperature, and at the metabolic level, CO_2_ uptake and internal CO_2_ concentrations displayed robust rhythms in similar conditions. Hartwell et al. (1999) reported circadian rhythms at the molecular level by showing 24 h rhythms in transcript abundances of one key CAM gene phosphoenolpyruvate carboxylase kinase. Furthermore, at the biochemical level, phosphorylation of another key CAM gene, phosphoenolpyruvate carboxylase, by phosphoenolpyruvate carboxylase kinase displays circadian rhythms in constant darkness (Nimmo et al., 1984). However, a thorough genomic investigation of circadian control in CAM plants has not been performed.

Identification and characterization of circadian-clock regulated genes requires measuring gene expression over a 24-h period (Bar-Joseph et al., 2012; Li et al., 2015; Hughes et al., 2017). Moreover, a large amount of time-course data, with 2- to 4-h sampling intervals, are currently available: *Arabidopsis thaliana* (Mockler et al., 2007), *Oryza sativa* (Filichkin et al., 2011*)*, *Populus trichocarpa* (Filichkin et al., 2011), *Solanum lycopersicum* (Higashi et al., 2016), *Brachypodium distachyon* (Koda et al., 2017), and *Ananas comosus* (Ming et al., 2015). Furthermore, numerous algorithms exist for detecting rhythmic expression within such data sets; however, different algorithms yield inconsistent results when used on a common data set (Keegan et al., 2007; Doherty and Kay, 2010; Deckard et al., 2013). For instance, the definition of “periodic” can vary between different algorithms, and depending on the algorithm used, can result in different genes being identified as rhythmic. Additionally, distributions of scores from periodicity detection algorithms are not bi-model, such that there is no clear distinction between periodic and non-periodic genes. Recently, a new approach for detection of rhythmic gene expression in time-course data was developed for integrating multiple rhythmic detection algorithms (Kelliher et al., 2016), allowing one to leverage multiple definitions of “periodic” as detailed by the different algorithms and arrive at “high confidence” rhythmic gene sets that don’t necessarily represent the only rhythmic genes. Applying such a method to C_3_ and CAM photosynthesis plants will provide insights into how the circadian clock propagates environmental signals across the two different photosynthetic types as well as provide clues to how CAM evolved from C_3_.

Therefore, the aim of this study is to investigate the conservation and diversification of rhythmic gene expression between a model C_3_ plant *A. thaliana* and a model obligate CAM plant *K. fedtschenkoi*. Through comparative analysis of time-course data using rhythmic detection algorithms (Deckard et al., 2013) we identified commonalities and differences in rhythmic genes between *A. thaliana* and *K. fedtschenkoi*. Our results provide further lines of evidence supporting the hypothesis that the circadian clock played a role in the evolution of CAM.

## Materials and Methods

### Time-Course Gene Expression Data

The diel expression data for *Kalanchoë fedtschenkoi* and *Arabidopsis thaliana* were obtained from Yang et al. (2017) and Mockler et al. (2007), respectively. *K. fedtschenkoi* genes with a max FPKM < 1 were removed. The *K. fedtschenkoi* expression data were collected at 2, 4, 6, 8, 10, 12, 14, 16, 18, 20, 22, and 24 h whereas the *A. thaliana* data were collected at 4, 8, 12, 16, 20, and 24 h after the initiation of the light period. Since the *A. thaliana* gene expression data were measured at 4-h intervals and the *K. fedtschenkoi* data were collected at 2-h intervals, the *A. thaliana* data were imputed to arrive at expression profiles for all *A. thaliana* and *K. fedtschenkoi* genes on the same time scale. Interpolation of the *A. thaliana* data for comparative gene expression analysis has been conducted before (Yang et al., 2017). In the present study, the piecewise cubic Hermite interpolating polynomial (pchip) interpolation function in the pandas Python library was used to interpolate the *A. thaliana* data at time points consistent with time intervals: 2, 4, 6, 8, 10, 12, 14, 16, 18, 20, 22, and 24 h. Pchip was used to maintain the shape of the data (Dong et al., 2011; Luo et al., 2014).

### Identify Rhythmic Genes in *K. fedtschenkoi* and *A. thaliana*

The rhythmic genes in *K. fedtschenkoi* and *A. thaliana* were identified by using a methodology developed by Kelliher et al. (2016), which applied four periodicity detection algorithms, i.e., de Lichtenberg (de Lichtenberg et al., 2005), JTK-CYCLE (JTK) (Hughes et al., 2010), Lomb-Scargle (Scargle, 1982) and persistent homology (Lomb, 1975), to the time-course gene expression data. The initial step in the overall method is to apply the periodicity detection algorithms to each dataset, summing the gene rankings output from each algorithm and reranking based on the cumulative ranks. We did not use the persistent homology step as it has been shown to give poor results when applied to higher organisms (Deckard et al., 2013). The second step in the Kelliher et al. (2016) method is to visually assess rhythmicity in the dataset of cumulative ranked genes. A 500 gene cohort was plotted to visually inspect where rhythmicity in expression decreased. A step size of 500 genes was empirically determined. A cutoff of 10,000 and 10,500 was used to identify top ranked genes for *A. thaliana* and *K. fedtschenkoi*, respectively. To validate this approach on time-course gene expression data with the length of one period, the top 10,000 ranked genes in *A. thaliana* were compared to a previously published rhythmic gene set using the same data and found a 48% overlap (Mockler et al., 2007). A 100% overlap was not expected because several factors such as data normalization, periodicity detection and/or algorithm score cutoff can result in varying sets of genes even from the same data set (Keegan et al., 2007; Kelliher et al., 2016).

We then applied a threshold based on the JTK algorithm at a *p* ≤ 0.06. Kelliher et al. (2016) based a threshold on the Lomb-Scargle algorithm but the JTK algorithm has been shown to give preference to cosine and peaked gene expression profiles (Deckard et al., 2013) and a previous study examining rhythmicity in transcription factors and transcription co-regulators in the CAM plant pineapple found cosine and peaked profiles were most successful in identifying cycling transcription factors and transcription co-regulators (Sharma et al., 2017).

### Functional Analysis of Rhythmic Genes *K. fedtschenkoi* and *A. thaliana*

Gene Ontology (GO) terms for the *K. fedtschenkoi* and *A. thaliana* genes were used from the gene annotation information downloaded from Phytozome v12.1 (Goodstein et al., 2012). *K. fedtschenkoi* genes encoding putative transcription factors were retrieved from Yang et al. (2017), while *A. thaliana* transcription factors (TFs) were retrieved from PlanTFDB v4.0 (Jin et al., 2017).

Using ClueGO (Bindea et al., 2009), observed GO biological process were subjected to the right-sided hypergeometric enrichment test at medium network specificity selection and *p*-value correction was performed using the Holm-Bonferroni step-down method (Holm, 1979). There was a minimum of 3 and a maximum of 8 selected GO-tree levels and each cluster was prescribed to include a minimum of between 3 and 4% of genes associated with each term. GO-term fusion and grouping settings were selected to minimize GO-term redundancy and the term enriched at the highest level of significance was used as the representative term for each functional cluster. The GO terms with *p*-values less than or equal to 0.05 were considered significantly enriched.

### Identification of Orthologous Genes

Ortholog groups consisting of proteins from 26 plant species were obtained from Yang et al. (2017). In short, ortholog groups were constructed using FastOrtho with default parameters selected, except for a BLASTp E-value cutoff of 1e-5 and an inflation value of 1.3. To determine if *K. fedtschenkoi* genes have homologs in other plant species, *K. fedtschenkoi* protein sequences were queried a local plant proteome database using BLASTp, with an E-value cutoff of 1e-10, implemented in the National Center for Biotechnology Information (NCBI) BLAST+ (Camacho et al., 2009). This local proteome database contained protein sequences from 83 plant species (Supplementary Table S3), including 70 species from PLAZA 4.0 (Van Bel et al., 2017) and 10 species from Phytozome 12.0 (Goodstein et al., 2012) and three *Agave* species with transcriptome sequenced (Gross et al., 2013; Abraham et al., 2016).

## Results

### Identifying Rhythmic Genes in *A. thaliana* and *K. fedtschenkoi*

**FIGURE 1:**
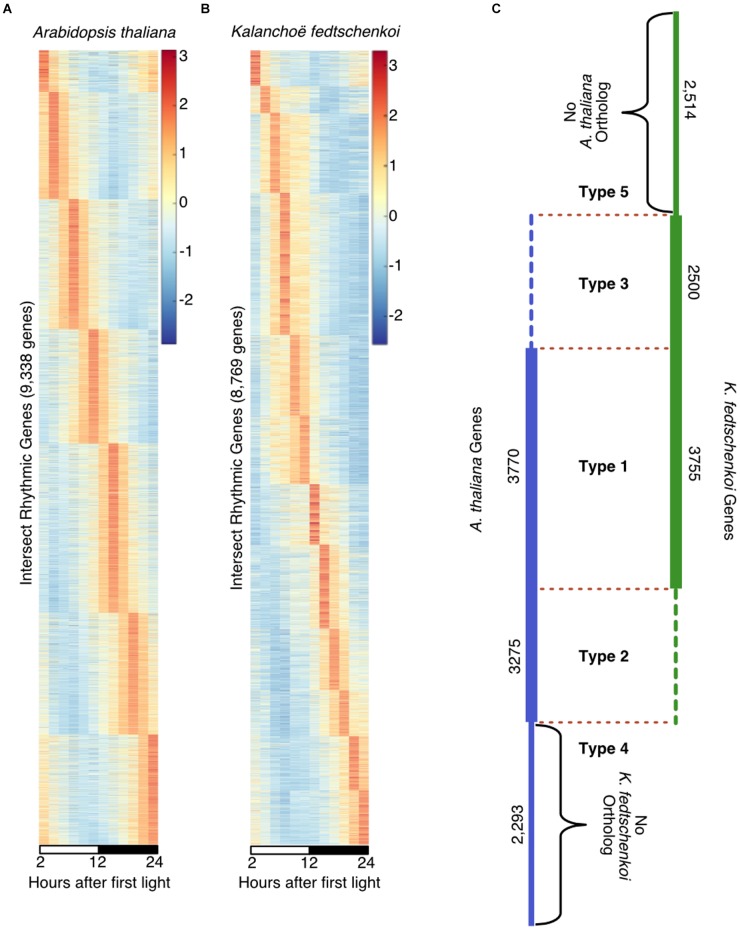
Rhythmically expressed genes in *Arabidopsis thaliana and Kalanchoë fedtschenkoi.* Taking the intersection of a visual inspection of cumulatively ranked genes and a JTK-CYCLE cutoff of 0.06 resulted in a qualitative selection of highly rhythmic genes in each species. **(A)** 9,338 genes were identified as highly rhythmic in *A. thaliana* indicated by the wave of peak expression through time. **(B)** 8,769 genes were identified as highly rhythmic in *Kalanchoë fedtschenkoi* as indicated by the wave of peak expression though time. White and black bars indicate daytime (12-h) and nighttime (12-h), respectively. The y-axes represent individual genes and transcript levels are depicted as a z-score change relative to mean expression for each gene, where values represent the number of standard deviations away from the mean. **(C)** Types of rhythmic orthologs and genes in *A. thaliana* (left) and *K. fedtschenkoi* (right). Type 1 refers to orthologs that are rhythmic in both species. Type 2 refers to orthologs that are rhythmic only in *A. thaliana*. Type 3 refers to orthologs that are rhythmic only in *K. fedtschenkoi*. Type 4 refers to genes found to be rhythmic only in *A. thaliana*. Type 5 refers to genes found to be rhythmic only in *K. fedtschenkoi*. Types are designated by their type names and separated by red dotted lines. Vertical numbers are the number of genes in each group from each plant species.

Using the Kelliher et al. (2016) method to identify “high confidence” rhythmic gene sets, 9,338 genes in *A. thaliana* (Figure 1A) and 8,769 genes in *K. fedtschenkoi* (Figure 1B) were estimated to be rhythmically expressed with a 24-h period, representing 34% of the genes in the *A. thaliana* genome and 28% of the genes in the *K. fedtschenkoi* genome. The expression patterns in Figures 1A,B reveal a continuum of phase-specific gene expression, which is a typical behavior of periodic genes controlled by the circadian clock in *A. thaliana* (Covington et al., 2008), *Solanum lycopersicum* (Higashi et al., 2016) and *Brachypodium distachyon* (Koda et al., 2017). Multiple criteria for evaluating expression patterns for individual genes in *A. thaliana* and *K. fedtschenkoi* in our study are provided in Supplementary Tables S1, S2.

### Functional Comparison of Rhythmic Genes Between *A. thaliana* and *K. fedtschenkoi*

A functional enrichment of both rhythmic gene sets was performed using ClueGO (Bindea et al., 2009) to gain insight into the physiological relevance of the circadian clock. Top functional groups enriched in the *A. thaliana* rhythmic gene set include pigment biosynthetic processes, ribonucleoprotein complex biogenesis and response to high light intensity, red or far red light, light stimulus and cold (Supplementary Table S4). The *K. fedtschenkoi* rhythmic gene set was enriched for organic acid metabolic processes, carboxylic acid metabolic processes, protein targeting to chloroplast, starch metabolic processes, oxylipin metabolic processes, phosphate-containing compound metabolic processes, photosynthesis (light harvesting), glyceraldehyde-3-phosphate metabolic process, isoprenoid biosynthetic process and plastid organization (Supplementary Table S4). Strikingly, no overlap in enriched functional groups was observed when comparing the dominate rhythmic processes between these two species.

**FIGURE 2:**
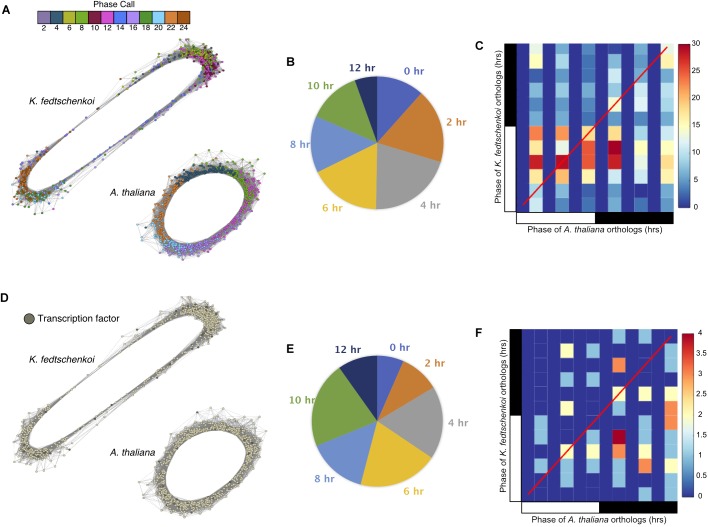
Gene co-expression networks of Type 1 ortholog groups containing only one rhythmic gene in each of the two plant species (*Arabidopsis thaliana* and *Kalanchoë fedtschenkoi)*. **(A)** Circular gene co-expression networks of rhythmic *A. thaliana* and *K. fedtschenkoi* orthologs. Genes are colored according to their phase call. **(B)** Pie chart categorizing the different phase shifts between *A. thaliana* and *K. fedtschenkoi* orthologs. **(C)** Heat map depicting the phase calls of orthologs between *A. thaliana* (x-axis) and *K. fedtschenkoi* (y-axis). The red line represents positions of identical phases between both species. The black and white bars indicated nighttime (12 h) and daytime (12 h), respectively. **(D)** Genes annotated as transcription factors in *A. thaliana* and *K. fedtschenkoi*. **(E)** Pie chart categorizing the different phase shifts between the transcription factor orthologs in *A. thaliana* and *K. fedtschenkoi*. **(F)** Heat map depicting the phase calls of transcription factor orthologs between *A. thaliana* (x-axis) and *K. fedtschenkoi* (y-axis). The red line represents positions of identical phases between both species. White and black bars indicate daytime (12-h) and nighttime (12-h), respectively.

To further investigate the physiological relevance of the circadian clock in a temporal context, a chronological gene co-expression network (GCN) was constructed from each rhythmic dataset, with subnetworks representing each phase of the circadian system (Supplementary Figure S2). Spearman’s rank correlation coefficient was calculated for all pair-wise combinations of the rhythmic genes in each dataset. The *A. thaliana* and *K. fedtschenkoi* GCN contained 9,338 rhythmic genes linked by 4,174,924 edges and 8,769 rhythmic genes linked by 4,590,438 edges, respectively, with a *r* ≥ 0.8 as a cutoff of co-expression. Visualizing phase calls of every gene with a color scheme, rhythmic genes can be seen chronologically connected in a circular network (Figure 2A), consistent with the diagonal stripe observed in the rhythmic gene expression heat maps for each species (Figures 1A,B). A similar result has been reported in a previous study in *B. distachyon* (Koda et al., 2017).

In the rhythmic gene sets for *A. thaliana* and *K. fedtschenkoi*, 644 and 923 genes were found to encode putative transcription factors (TFs), respectively. Consistent with the previous study (Koda et al., 2017), we found that the rhythmic expression of genes is correlated to transcription factors that were also rhythmically expressed at the transcript level. Rhythmic TFs in both species were found in all phases of the day and night (Supplementary Figure S2) and only photosynthesis and starch metabolism were common to both species’ rhythmic gene lists (Supplementary Table S5).

To evaluate the conservation of rhythmicity and phase of expression between *A. thaliana* and *K. fedtschenkoi*, ortholog groups were constructed using the protein sequences from these two species (Figure 1C). Between the rhythmic gene sets, five types of orthologs and genes were identified: Type 1 containing orthologs that are rhythmic in both species, Type 2 containing orthologs that are rhythmic only in *A. thaliana*, Type 3 containing orthologs that are rhythmic only in *K. fedtschenkoi*, Type 4 containing genes that are rhythmic and found only in *A. thaliana*, and Type 5 containing genes that are rhythmic and found only in *K. fedtschenkoi*. Gene lists for each species in each ortholog group type can be found in Supplementary Table S6. Gene numbers and functional investigations in each type are described below.

### Type 1: Rhythmic Ortholog Groups Shared Between *A. thaliana* and *K. fedtschenkoi*

2,641 ortholog groups were identified to contain at least one rhythmic gene in each of the two plant species analyzed here. GO enrichment of each subset of Type 1 rhythmic genes displayed a 37% overlap in enriched biological processes, including protein phosphorylation, organic acid metabolic process, transport, photosynthesis and generation of precursor metabolites and energy (Supplementary Table S7). In general, cross-species identification of rhythmic orthologs can be challenging due to “many-to-many” orthologous relationships and when an ortholog group contains genes which are rhythmically expressed in only one of the two species. Therefore, further analysis of conserved rhythmicity within the Type 1 group was limited to only those ortholog groups displaying a single expression profile within a species. This restriction resulted in 768 ortholog groups between the two species where both species’ genes were rhythmic. Rhythmic genes were observed in all phases of the day and night that were sampled in each species (Figure 2A). Several key CAM genes involved in CO_2_ fixation in *K. fedtschenkoi* (Yang et al., 2017) were found to be rhythmic, as well as several of their *A. thaliana* orthologs (Supplementary Tables S8, S9). Evidence of both synchronization and desynchronization were found in one-to-one ortholog groups, e.g., with the NADP-malic enzyme (Supplementary Table S9).

To further understand the functional relevance of the rhythmic genes, phase shifts between rhythmic orthologs in the two species were examined next. Phase shifts are defined here as the difference between two rhythmic genes’ phase calls, which is the time point where maximum transcript abundance occurs for a gene. We define two orthologs to be *synchronized* if their phase calls are within 4 h of each other and *unsynchronized* otherwise. Of the 768 ortholog groups, 386 (50%) ortholog groups contained ortholog pairs with synchronized gene expression (Figure 2B), with several synchronized ortholog pairs phased between morning and evening (Figure 2C). The second sub-type, unsynchronized orthologous gene pairs, contained 382 (50%) ortholog groups (Figure 2B). One group of *K. fedtschenkoi* genes peaking at midday had orthologs in *A. thaliana* peaking in the morning, while another group of *K. fedtschenkoi* genes peaking at midday had orthologs in *A. thaliana* peaking just after dusk, suggesting differential regulation of rhythmic orthologous gene pairs between *A. thaliana* and *K. fedtschenkoi*. TFs were also found in the 768 ortholog groups and in all phases of the day and night in each species (Figure 2D). Of the 61 TF ortholog groups, 40 (66%) contained unsynchronized TF ortholog pairs (Figure 2E). The 61 orthologous TFs in *A. thaliana* and *K. fedtschenkoi* are listed in Table 1, along with the respective *A. thaliana* gene annotation. Several orthologous TFs had similar phase shifts as the two groups of differential regulated orthologs described earlier (Figure 2F), potentially accounting for the difference in gene expression between orthologous gene pairs.

**Table 1:** Type 1 rhythmic orthologous transcription factors in *Kalanchoë fedtschenkoi* and *Arabidopsis thaliana*.

*K. fedtschenkoi*		*A. thaliana*			Phase shift (hours)
Gene locus	Phase call	Gene locus	Phase call	Description	
Kaladp0057s0097	2	AT1G09530	20	Phytochrome interacting factor 3	6
Kaladp0015s0032	4	AT4G38890	16	FMN-linked oxidoreductases superfamily protein	12
Kaladp0003s0081	6	AT5G42200	12	RING/U-box superfamily protein	6
Kaladp0011s0211	6	AT3G45880	16	2-oxoglutarate (2OG) and Fe (II)-dependent oxygenase superfamily protein	10
Kaladp0098s0059	6	AT1G76710	20	SET domain group 26	10
Kaladp0087s0172	6	AT4G00090	20	Transducin/WD40 repeat-like superfamily protein	10
Kaladp0053s0598	6	AT5G64730	20	Transducin/WD40 repeat-like superfamily protein	10
Kaladp0024s0982	6	AT2G47450	24	Chloroplast signal recognition particle component	6
Kaladp0058s0485	6	AT5G51110	24	Transcriptional coactivator/pterin dehydratase	6
Kaladp0039s0495	8	AT1G68550	16	Integrase-type DNA-binding superfamily protein	8
Kaladp0095s0706	8	AT1G75430	16	BEL1-like homeodomain 11	8
Kaladp0037s0533	8	AT5G28300	16	Duplicated homeodomain-like superfamily protein	8
Kaladp1129s0043	8	AT4G17060	20	FRIGIDA interacting protein 2	12
Kaladp0095s0494	8	AT4G32570	20	TIFY domain protein 8	12
Kaladp0050s0018	8	AT5G28640	24	SSXT family protein	8
Kaladp0034s0058	10	AT1G10610	4	Basic helix-loop-helix (bHLH) DNA-binding superfamily protein	6
Kaladp0068s0095	10	AT4G12240	16	Zinc finger (C2H2 type) family protein	6
Kaladp0809s0115	10	AT4G29940	16	Pathogenesis related homeodomain protein A	6
Kaladp0042s0203	10	AT5G14370	16	CCT motif family protein	6
Kaladp0081s0357	10	AT5G63420	16	RNA-metabolizing metallo-beta-lactamase family protein	6
Kaladp0039s0570	10	AT3G52190	20	Phosphate transporter traffic facilitator1	10
Kaladp0044s0029	10	AT2G32000	24	DNA topoisomerase, type IA, core	10
Kaladp0808s0018	12	AT1G22860	4	Vacuolar sorting protein 39	8
Kaladp0033s0181	12	AT3G18640	20	Zinc finger C-x8-C-x5-C-x3-H type family protein	8
Kaladp0015s0185	12	AT1G49040	24	stomatal cytokinesis defective/SCD1 protein	12
Kaladp0099s0116	12	AT5G50970	24	Transducin family protein/WD-40 repeat family protein	12
Kaladp0085s0129	12	AT5G56780	24	Effector of transcription2	12
Kaladp0004s0037	14	AT2G04240	8	RING/U-box superfamily protein	6
Kaladp0746s0006	14	AT2G47270	8	Transcription factor UPBEAT protein	6
Kaladp0011s0744	14	AT1G08810	24	myb domain protein 60	10
Kaladp0008s0534	14	AT2G39810	24	Ubiquitin-protein ligase	10
Kaladp0055s0201	14	AT4G37650	24	GRAS family transcription factor	10
Kaladp0093s0080	16	AT4G00150	24	GRAS family transcription factor	8
Kaladp0048s0281	16	AT5G01160	24	RING/U-box superfamily protein	8
Kaladp0060s0155	18	AT3G50590	8	Transducin/WD40 repeat-like superfamily protein	10
Kaladp0011s0085	18	AT1G58025	12	DNA-binding bromodomain-containing protein	6
Kaladp0050s0333	22	AT1G68190	8	B-box zinc finger family protein	10
Kaladp0048s0675	22	AT3G23490	8	Cyanase	10
Kaladp0011s0272	22	AT5G03720	12	Heat shock transcription factor A3	10
Kaladp0036s0299	24	AT5G58410	16	HEAT/U-box domain-containing protein	8

*“Phase Call” is the hours after first light which the respective gene peaked in expression. “Phase Shift” is the number of h the K. fedtschenkoi gene expression profile shifted from its A. thaliana ortholog’s gene expression profile. The shift was calculated by subtracting the phase calls of each ortholog.*

### Type 2: Rhythmic *A. thaliana* Genes With Arrhythmic Orthologs in *K. fedtschenkoi*

**FIGURE 3:**
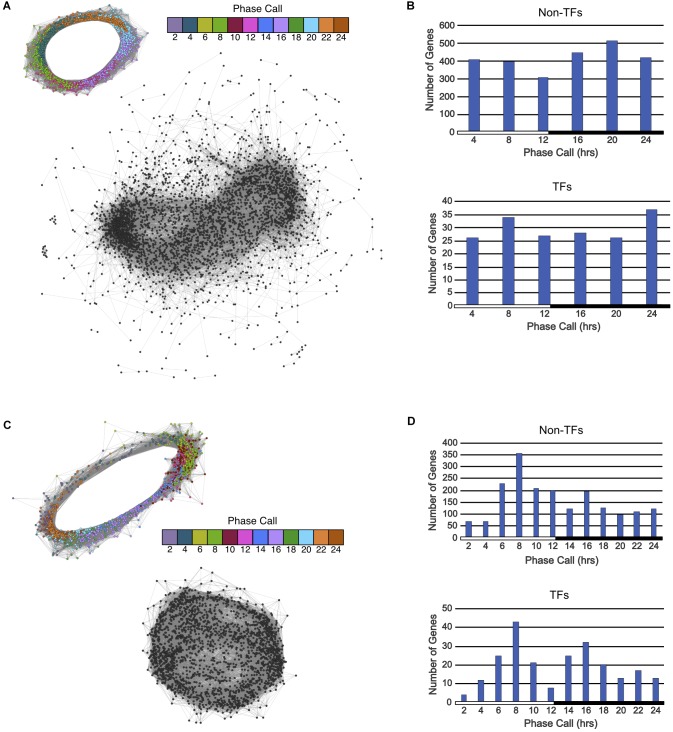
Gene co-expression networks and phase call distributions of Type 2 and Type 3 ortholog groups described in Figure 1C. **(A)** Circular gene co-expression networks of rhythmic genes in *A. thaliana* (top) and arrhythmic genes in *K. fedtschenkoi* (bottom). Genes are colored according to their phase call. **(B)** Phase call distributions of rhythmic *A. thaliana* non-TF genes (top) and rhythmic *A. thaliana* TF genes (bottom). **(C)** Circular gene co-expression networks of rhythmic genes in *K. fedtschenkoi* (top) and arrhythmic genes in *A. thaliana* (bottom) orthologs. Genes are colored according to their phase call. **(D)** Phase call distributions of rhythmic *K. fedtschenkoi* non-TF genes (top) and rhythmic *K. fedtschenkoi* TF genes (bottom). White and black bars indicate daytime (12-h) and nighttime (12-h), respectively

2,797 ortholog groups were identified that had at least one *A. thaliana* gene that was rhythmic and all *K. fedtschenkoi* genes were arrhythmic. A total of 3,275 *A. thaliana* genes found to be rhythmic that had no rhythmic *K. fedtschenkoi* orthologs (Figure 1C). Only two biological processes were enriched in this *A. thaliana* gene set: cellular nitrogen compound metabolism and response to endoplasmic reticulum stress (Supplementary Table S7). To further examine differential rhythmicity within Type 2 ortholog groups, ortholog groups that also contained arrhythmic *A. thaliana* genes were filtered out, resulting in 2,258 ortholog groups that only contained rhythmic *A. thaliana* and arrhythmic *K. fedtschenkoi* genes. Clustering of the arrhythmic *K. fedtschenkoi* genes did not display a circular network typical of rhythmic genes, further validating the rhythmic gene expression detection method used in this study (Figure 3A). A slightly higher number of rhythmic *A. thaliana* genes were found with phase calls occurring during the night (Figure 3B). Within these 2,258 ortholog groups, 178 rhythmic *A. thaliana* TFs were identified and were phased to all phases of the day and night (Figure 3B).

### Type 3: Rhythmic *K. fedtschenkoi* Genes With Arrhythmic Orthologs in *A. thaliana*

A total of 2,500 *K. fedtschenkoi* rhythmic genes were found to have no rhythmic *A. thaliana* orthologs in their respective ortholog groups (Figure 1C). Numerous processes were enriched in the *K. fedtschenkoi* gene set, including several metabolic processes and transcription from plastids (Supplementary Table S7). Differential rhythmicity was further examined and resulted in 1,759 ortholog groups that only contained rhythmic *K. fedtschenkoi* and arrhythmic *A. thaliana* genes. Arrhythmic *A. thaliana* genes did not cluster into a circular pattern as their rhythmic *K. fedtschenkoi* orthologs did (Figure 3C). Plotting the distribution of phase calls of the rhythmic *K. fedtschenkoi* genes revealed a concentration of genes around midday (Figure 3D). Within this set of ortholog groups, 233 *K. fedtschenkoi* TFs were identified, which were phased to all phases of the day and night and displayed a bimodal distribution of phase calls (Figure 3D).

### Type 4: Rhythmic *A. thaliana* Genes Only Without Orthologs in *K. fedtschenkoi*

788 ortholog groups were identified that had at least one rhythmic *A. thaliana* gene, totaling 2,293 rhythmic genes in *A. thaliana*, with no putative orthologs in *K. fedtschenkoi* (Figure 1C). Only toxin metabolic process was enriched in this gene set (Supplementary Table S7).

### Type 5: Rhythmic *K. fedtschenkoi* Genes Without Orthologs in *A. thaliana*

There were 2,514 predicted rhythmic genes in *K. fedtschenkoi* with no detected orthologs in *A. thaliana* (Figure 1C). Only monoterpenoid biosynthetic process was enriched in this gene set (Supplementary Table S7). To determine if any genes in this gene set could be CAM-specific (i.e., shared by multiple CAM species but not by other non-CAM species) or *K. fedtschenkoi*-specific (i.e., not shared by other CAM and non-CAM species), protein sequences for each gene were BLASTed against a collection of plant proteomes spanning monocots and dicots and including all photosynthetic types (i.e., C_3_, C_4,_ and CAM) (Supplementary Table S3). Results indicate that 1,486 *K. fedtschenkoi* genes in this gene set were found in both non-CAM and CAM species (1 < e-10; Supplementary Table S10). Four *K. fedtschenkoi* genes were found to have homologs in only other CAM species and 443 *K. fedtschenkoi* genes were found to have homologs in only the non-CAM species. Of the four genes found in only CAM species, none were found in all of the six CAM species tested. Finally, 581 genes were found to be *K. fedtschenkoi*-specific. A majority of the rhythmic *K. fedtschenkoi*-specific genes had phase calls during midday, approximately 8 h after the beginning of light period (Supplementary Table S11), which coincided with the four CAM-specific *K. fedtschenkoi* genes (Supplementary Table S11).

### Core Clock Genes in *A. thaliana* and *K. fedtschenkoi*

Investigating gene expression between *A. thaliana* genes and their orthologs in *K. fedtschenkoi* revealed cases of loss and gain of rhythmicity as well as phase shifts. One mechanism that can cause differences in gene expression between orthologs is differences between the regulatory network an ortholog is connected to. For example, through evolutionary processes, an ortholog can become connected to a different TF(s) in the network or become connected to an entirely different regulatory network, via reconnection with a new TF(s) or the its original TF(s) connected with a new network (Nadimpalli et al., 2015; Mateos et al., 2017). Both of these mechanisms can result in altered gene expression of an ortholog. The core circadian clock, which is a small regulatory network of interacting TFs, is known as a core mechanism that drives rhythmic gene expression in plants (Nohales and Kay, 2016). Therefore, we investigated the gene expression of core circadian clock TFs to determine if the loss and gain of rhythmicity and phase shifts seen above could be a result of changes in the core circadian clock network, e.g., phase shifts of or loss of rhythmicity in gene expression of core circadian clock TFs.

**FIGURE 4:**
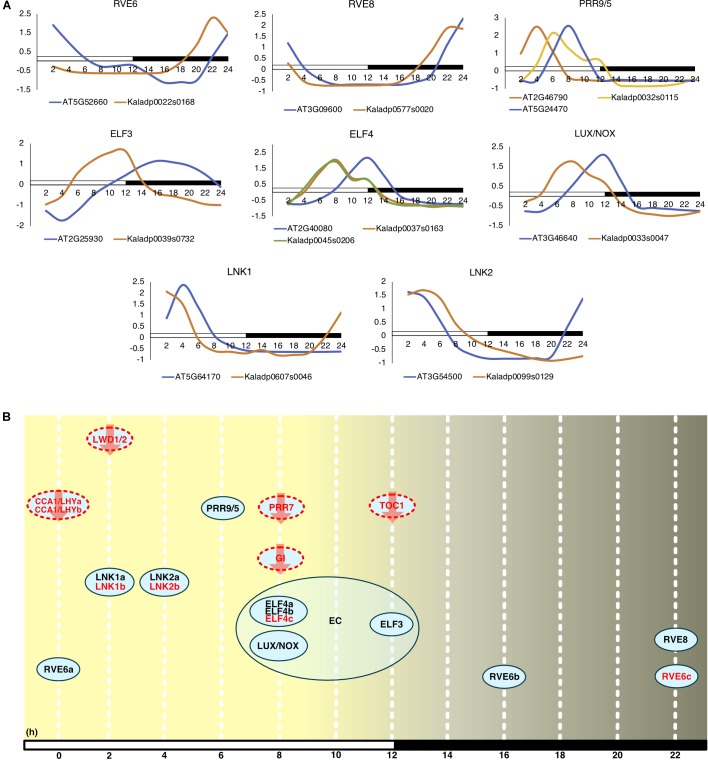
Expression profiles of *Arabidopsis thaliana* core clock genes and their *Kalanchoe fedtschenkoi* orthologs. **(A)** Expression of clock orthologs where the *K. fedtschenkoi* gene had circadian-like expression, as described in the text. The *A. thaliana* gene names starts with “AT” and the *K. fedtschenkoi* gene names starts with “Ka.” X-axis corresponds to number of hours after first light. Time points 2 and 14 correspond to subjective dawn and dusk, respectively. Transcript levels are depicted as a z-score change relative to mean expression for each gene, where values represent the number of standard deviations away from the mean. **(B)** Phase of day for core clock orthologs in *K. fedtschenkoi*. Ovals represent core clock genes. Red text and arrows indicate the gene does not have either an absolute amplitude ≥ 10, fold-change ≥ 2 and/or a JTK-CYCLE *p* ≤ 0.05. Genes with a red dotted outline indicates that the timing of their expression, as indicated here, is based on the phase call of their *A. thaliana* ortholog. The numbers arrayed at the bottom of the image indicate the number of hours passed after first light. EC, Evening complex. White and black bars indicate daytime (12-h) and nighttime (12-h), respectively.

Commonly, the *A. thaliana* core clock model (Nohales and Kay, 2016) is used to infer conservation of clock genes in other plant species. Following this, orthologs of the clock genes in *A. thaliana* were found in *K. fedtschenkoi* (Table 2 and Supplementary Table S12). Variation in the *K. fedtschenkoi* core clock network was observed when assaying each component’s expression dynamics. Core clock genes are typically characterized by cardinal circadian parameters, such as high amplitudes and fold-changes, along with highly statistically significant rhythmicity (Doherty and Kay, 2010; Zhang et al., 2014; Hughes et al., 2017). Using high amplitudes (max_expr_-min_expr_) (FPKM units) as absolute amplitudes > 10, high fold-changes (max_expr_/min_expr_) (FPKM units) as fold-changes > 2 and statistically rhythmic as JTK *p* ≤ 0.05, only 10 of the 23 *K. fedtschenkoi* core clock orthologs were found (Table 2). Within these 10 genes, there were paralogs of RVE6 and ELF4. Interestingly, ELF3, ELF4, and LUX, which make up the evening complex (Nusinow et al., 2011) of the circadian clock network, displayed a concerted phase shift of 4 h ahead of their *A. thaliana* orthologs (Figure 4A). Of the remaining 13 core clock orthologs, four did not pass the gene expression threshold (max FPKM > 1) used before the analysis of gene expression rhythmicity (Table 2). Constructing the core clock model for *K. fedtschenkoi* with this information could suggest a different architecture of the clock (Figure 4B).

**Table 2:** Circadian parameters of *K. fedtschenkoi* core clock orthologs.

Gene name	Gene locus	Normalized rank	Mean FPKM	Absolute amplitude	Fold-change	JTK-CYCLE *p*-value
LUX/NOX	Kaladp0033s0047	34	19.45	42.12	10.95	1.06E-05
ELF3	Kaladp0039s0732	177	30.61	64.15	11.13	5.39E-04
ELF4a	Kaladp0037s0163	207	11.75	35.32	47.72	4.84E-05
ELF4b	Kaladp0045s0206	737	36.91	122.38	622.21	1.46E-03
PRR9/5	Kaladp0032s0115	818	36.87	127.70	327.39	4.84E-05
RVE8	Kaladp0577s0020	1180	40.88	143.53	992.09	4.84E-05
RVE6a	Kaladp0019s0045	2589	52.55	76.76	3.73	7.92E-03
RVE6b	Kaladp0022s0168	2672	154.49	700.15	476.50	4.84E-05
LNK1a	Kaladp0607s0046	4139	13.85	45.15	33.56	1.65E-02
LNK2a	Kaladp0099s0129	4214	41.61	99.10	15.51	3.22E-02
RVE6c	Kaladp0055s0349	5756	13.42	8.30	1.97	1.65E-02
ELF4c	Kaladp0059s0037	6111	6.34	7.77	3.67	3.22E-02
LNK2b	Kaladp0060s0264	6361	60.40	124.40	11.48	1.05E-01
GI	Kaladp0040s0489	6902	4.18	9.37	6.60E+07	2.89E-01
LNK1b	Kaladp0047s0123	12058	3.17	14.92	6.81E+05	1.00E+00
PRR7	Kaladp0001s0237	12078	0.74	3.23	NA	6.92E-01
CCA/LHYa	Kaladp0066s0115	13093	1.74	8.30	3.97E+07	4.54E-01
CHE	Kaladp0032s0054	14327	23.19	22.66	2.54	2.89E-01
LWD1/2	Kaladp0048s0797	15008	13.89	4.12	1.34	4.54E-01
TOC1	Kaladp0040s0446	NA	0.18	0.67	26665.50	NA
PRR7	Kaladp0101s0041	NA	0.22	0.63	2.19E+43	NA
CCA/LHYb	Kaladp0496s0018	NA	0.10	0.58	2.85E+23	NA

## Discussion

CAM plants arose independently from diverse C_3_ ancestors and developed two distinguishing features, strict temporal control of metabolism (e.g., CO_2_ fixation, sugar accumulation) and temporally inverted stomatal movement patterns relative to C_3_ plants. The temporal significance of these features has led to the hypothesis that these features developed under the control of the circadian clock (Boxall et al., 2005; Cushman et al., 2008). To further test this hypothesis, we first identified rhythmic genes in a CAM and C_3_ species and identified orthologs between the two species. We next examined the expression profiles looking for evidence of phase shifts between rhythmic orthologs in these two species. In this study, we revealed diversification of circadian rhythmicity between CAM and C_3_ photosynthesis species. Moreover, differences were found between the *K. fedtschenkoi* core clock network and the *A. thaliana* model core clock network (Table 2), providing molecular evidence supporting the hypothesis that the core clock could have impacts on CAM evolution.

Approximately 30% of all genes were found to be rhythmically expressed in both species, which is consistent with previous circadian studies grown under similar conditions and using whole-leaf samples (Michael et al., 2008; Filichkin et al., 2011). Prior studies have examined rhythmic transcriptomes of various plant species and among them, similar functional groups have been found enriched in their plant’s respective rhythmic gene set (Harmer et al., 2000; Filichkin et al., 2011; Koda et al., 2017). Surprisingly, there was no overlap in enriched functional groups when comparing the entire *A. thaliana* and *K. fedtschenkoi* rhythmic gene sets to each other. Our analysis cannot explicitly explain why there is no overlap in enriched functional groups. However, a potential explanation could be that the circadian clock controls alternate biological processes in *K. fedtschenkoi* in response to extreme environmental fluctuations. For example, CAM plants typically experience more drastic temperature differences between night and day than their C_3_ counterparts. Additionally, temperature perception by the clock has been found to be different between plants resulting in different regimes of genes being transcribed and therefore different biological processes to occur (Filichkin et al., 2011). It would be worth exploring how C_3_ plants respond at the transcriptional level to similar temperature conditions to determine if any parallels exist which could give insight into the CAM-to-C_3_ transition.

To determine if an increase in the temporal scale could further reveal similarities and differences in rhythmic biological processes, we examined phase specificity of biological processes. Photosynthesis and carbohydrate metabolism are plant processes that take place during the daylight hours and the circadian clock is known to play a crucial role in their regulation (Adams and Carre, 2011; Filichkin et al., 2011; Koda et al., 2017). Consistent with this, starch metabolism and photosynthesis were both enriched during midday for both species, in turn confirming conservation of these two pathways across C_3_ and CAM species. Though the similarities cease there and the idea of the clock controlling alternative biological processes is further supported as several processes are phase-specifically enriched in *K. fedtschenkoi* and not in *A. thaliana*. For instance, organic acid synthesis was enriched in the rhythmic transcriptome of *K. fedtschenkoi*, in line with the strict schedule of CAM-related metabolism to prevent futile cycling between dark-period CO_2_ fixation to malate and light-period malate decarboxylation (Borland et al., 2014). Furthermore, processes related to gluconeogenesis were found enriched in *K*. *fedtschenkoi*’s rhythmic transcriptome and not in *A. thaliana*’s rhythmic transcriptome. Wai et al. (2017) have recently shown that metabolic processes such as glycolysis and gluconeogenesis have day-night rhythms in the CAM plant pineapple. These results support the idea that maintaining metabolic homeostasis via strict scheduling of the associated genes is a defining feature of CAM plants.

Several studies have compared rhythmic gene sets between different species and found conserved core clock networks but divergent clock output networks (Keegan et al., 2007; Michael et al., 2008; Filichkin et al., 2011; Eckel-Mahan et al., 2013; Boyle et al., 2017; Yin et al., 2018). Our comparative analysis of rhythmic gene sets between *A. thaliana* and *K. fedtschenkoi* also revealed divergence in clock output networks, but also in the core clock network (Figure 4). Focusing on clock output first, two clock-related features, which were observed in Type 1 and 2 ortholog groups, account for the divergence in clock output. The first clock-related feature is of synchronization of rhythmic orthologs. These synchronized orthologs were enriched with functional groups related to photosynthetic processes and a large portion of these orthologs were found phased during the day, coinciding with when photosynthesis takes place in both species (Brautigam et al., 2017). These results are comparable with the results of phase-specific enrichment of functional groups across the entire rhythmic gene sets of both species, providing further support that the circadian clock plays an important role in photosynthesis in both C_3_ and CAM species.

The second clock-related feature is conserved rhythmicity between orthologs but divergence in timing of expression. The TFs in Table 1 represent potential mechanisms by which the clock alters rhythmic gene expression in the context of CAM evolution beyond just CO_2_ fixation. Specifically, the listed TFs could be associated with different core clock genes in *K. fedtschenkoi* than in *A. thaliana*, thus shifting not only their own gene expression but the genes they regulate. Several key CAM-related CO_2_ fixation genes were found in the Type 1 ortholog groups that displayed this clock phenotype, although, none were found in the two groups of phase-shifted orthologs and TFs. However, this does not rule out the remaining TFs displaying a different phase shift potentially being the mechanism behind the phase shift of these key CAM-related CO_2_ fixation genes. Regardless, the TFs in Table 1 warrant further functional investigation into how they are integrated into the output of the clock between species and what downstream processes they regulate.

The core clock network is one of the main causes of rhythmic gene expression seen in plants and has typically been found conserved across plant species (Filichkin et al., 2011; Koda et al., 2017; Sharma et al., 2017). In *K. fedtschenkoi*, several genes orthologous to *A. thaliana* core clock genes were found conserved in copy number and expression dynamics (Figure 4). However, some differences were observed between components of each species’ core clock network. For example, the evening complex, consisting of ELF4, ELF3, and LUX, was found to be altered in gene copy number (i.e., ELF4) and in timing of gene expression (Figure 4). Variation in gene copy numbers between orthologous core clock genes is not surprising as it has been observed in other species, the mechanism likely being fractionation and/or loss of genes during speciation events (Lou et al., 2012). The shifting of expression in the components of the evening complex, mostly to midday (Figure 4), does present an inserting case, as a common theme seen in this comparative analysis was rhythmic *K. fedtschenkoi* genes, including TFs, being highly phased to midday (Figures 2, 3). Whether the shift in the evening complex is cause for the joint shift in rhythmic genes seen here cannot be determined in this study. However, the fact that other core clock genes did not display a similar shift in gene expression and rhythmic gene expression is typically driven by the core clock does give reason for further investigation into this potential relationship. Studies examining potential relationships between TFs in Table 1 and components of the evening complex would provide better insight into how the circadian clock played a role in the evolution of CAM.

For a further example, some components, such as CCA1 and TOC1, were found to have low expression (Table 2), which is not typical of core clock genes (Lou et al., 2012; Yeung et al., 2018). This study is unable to identify the mechanism(s) behind the low expression values of these core clock genes; however, a possible cause could be feedback from clock output networks back into the core clock network. Feedback regulation between the clock and input signals as well as between output networks and the clock is a common feature of the circadian system (Fankhauser and Staiger, 2002; Li et al., 2011; Wenden et al., 2011; Chow et al., 2014; Kolmos et al., 2014). The low expression of some core clock genes could be a gating and/or compensation mechanism by the clock to some unknown factor(s). Alternatively, these plants were grown in diel conditions, so the impact of environmental variations, such as light, cannot be exclude as causes. To further investigate differential rhythmicity of *K. fedtschenkoi* core clock components with their respective *A. thaliana* orthologs, it will be necessary to generate time-course transcriptome-sequencing data for leaf samples collected from both *A. thaliana* and *K. fedtschenkoi* at 2-h intervals during 48-h continuous light or dark period. A comprehensive analysis of time-course transcriptome-sequencing data, such as those described in Michael et al. (2008) and Filichkin et al. (2011), from *A. thaliana* and *K. fedtschenkoi* leaves under various light and temperature conditions would provide further insight in circadian control of CAM. Additionally, it was recently hypothesized that post-transcriptional regulation plays an important role in circadian clocks because a transcript does not oscillate does not mean that the protein levels are not rhythmic (Kojima et al., 2011; Lim and Allada, 2013). Future studies on rhythmic profiles of protein expression will be needed to gain a comprehensive understanding of circadian rhythm in plants.

## Data Availability

All datasets generated for this study are included in the manuscript and the Supplementary Files.

## Author Contributions

RoM and XY conceived the research. RoM performed all data the analyses and wrote the manuscript. RiM, FM, SH, GT, and XY provided input during the study and edited the manuscript.

## Conflict of Interest Statement

The authors declare that the research was conducted in the absence of any commercial or financial relationships that could be construed as a potential conflict of interest.

## References

[B1] Abraham P. E. Yin H. Borland A. M. Weighill D. Lim S. D. De Paoli H. C. (2016). Transcript, protein and metabolite temporal dynamics in the CAM plant Agave. *Nat Plants* 2 16178. 10.1038/nplants.2016.178 27869799

[B2] Adams S. Carre I. A. (2011). Downstream of the plant circadian clock: output pathways for the control of physiology and development. *Essays Biochem.* 1 53–69. 10.1042/BSE0490053 21819384

[B3] Bar-Joseph Z. Gitter A. Simon I. (2012). Studying and modelling dynamic biological processes using time-series gene expression data. *Nat. Rev. Genet.* 13 552–564. 10.1038/nrg3244 22805708

[B4] Bell-Pederson D. Cassone V. M. Earnest D. J. Golden S. S. Hardin P. E. Thomas T. L. (2005). Circadian Rhythms from Multiple Oscillators: Lessons From Diverse Organisms. *Nature Reviews: Genetics* 6 544–556. 10.1038/nrg1633 15951747 PMC2735866

[B5] Bindea G. Mlecnik B. Hackl H. Charoentong P. Tosolini M. Kirilovsky A. (2009). ClueGO: a Cytoscape plug-in to decipher functionally grouped gene ontology and pathway annotation networks. *Bioinformatics* 25 1091–1093. 10.1093/bioinformatics/btp101 19237447 PMC2666812

[B6] Borland A. M. Hartwell J. Weston D. J. Schlauch K. A. Tschaplinski T. J. Tuskan G. A. (2014). Engineering crassulacean acid metabolism to improve water-use efficiency. *Trends Plant Sci.* 19 327–338. 10.1016/j.tplants.2014.01.006 24559590 PMC4065858

[B7] Boxall S. F. Foster J. M. Bohnert H. J. Cushman J. C. Nimmo H. G. Hartwell J. (2005). Conservation and divergence of circadian clock operation in a stress-inducible Crassulacean acid metabolism species reveals clock compensation against stress. *Plant Physiol.* 137 969–982. 10.1104/pp.104.054577 15734916 PMC1065398

[B8] Boyle G. Richter K. Priest H. D. Traver D. Mockler T. C. Chang J. T. (2017). Comparative Analysis of Vertebrate Diurnal/Circadian Transcriptomes. *PLoS ONE* 12:e0169923. 10.1371/journal.pone.0169923 28076377 PMC5226840

[B9] Brautigam A. Schluter U. Eisenhut M. Gowik U. (2017). On the Evolutionary Origin of CAM Photosynthesis. *Plant Physiol.* 174 473–477. 10.1104/pp.17.00195 28416703 PMC5462059

[B10] Camacho C. Coulouris G. Avagyan V. Ma N. Papadopoulos J. Bealer K. (2009). BLAST+: architecture and applications. *BMC Bioinformatics* 10:421. 10.1186/1471-2105-10-421 20003500 PMC2803857

[B11] Chow B. Y. Sanchez S. E. Breton G. Pruneda-Paz J. L. Krogan N. T. Kay S. A. (2014). Transcriptional regulation of LUX by CBF1 mediates cold input to the circadian clock in Arabidopsis. *Curr. Biol.* 24 1518–1524. 10.1016/j.cub.2014.05.029 24954045 PMC4090264

[B12] Covington M. F. Maloof J. N. Straume M. Kay S. A. Harmer S. L. (2008). Global transcriptome analysis reveals circadian regulation of key pathways in plant growth and development. *Genome Biol.* 9 R130. 10.1186/gb-2008-9-8-r130 18710561 PMC2575520

[B13] Cushman J. C. Tillett R. L. Wood J. A. Branco J. M. Schlauch K. A. (2008). Large-scale mRNA expression profiling in the common ice plant, Mesembryanthemum crystallinum, performing C3 photosynthesis and Crassulacean acid metabolism (CAM). *J. Exp. Bot.* 59 1875–1894. 10.1093/jxb/ern008 18319238

[B14] de Lichtenberg U. Jensen L. J. Fausboll A. Jensen T. S. Bork P. Brunak S. (2005). Comparison of computational methods for the identification of cell cycle-regulated genes. *Bioinformatics* 21 1164–1171. 10.1093/bioinformatics/bti093 15513999

[B15] Deckard A. Anafi R. C. Hogenesch J. B. Haase S. B. Harer J. (2013). Design and analysis of large-scale biological rhythm studies: a comparison of algorithms for detecting periodic signals in biological data. *Bioinformatics* 29 3174–3180. 10.1093/bioinformatics/btt541 24058056 PMC4471443

[B16] Doherty C. J. Kay S. A. (2010). Circadian control of global gene expression patterns. *Annu. Rev. Genet.* 44 419–444. 10.1146/annurev-genet-102209-163432 20809800 PMC4251774

[B17] Dong B. Zhang P. Chen X. Liu L. Wang Y. He S. (2011). Predicting Housekeeping Genes Based on Fourier Analysis. *PLoS ONE* 6:e21012. 10.1371/journal.pone.0021012 21687628 PMC3110801

[B18] Eckel-Mahan K. L. Patel V. R. de Mateo S. Orozco-Solis R. Ceglia N. J. Sahar S. (2013). Reprogramming of the circadian clock by nutritional challenge. *Cell* 155 1464–1478. 10.1016/j.cell.2013.11.034 24360271 PMC4573395

[B19] Fankhauser C. Staiger D. (2002). Photoreceptors in Arabidopsis thaliana: light perception, signal transduction and entrainment of the endogenous clock. *Planta* 216 1–16. 10.1007/s00425-002-0831-4 12430009

[B20] Filichkin S. A. Breton G. Priest H. D. Dharmawardhana P. Jaiswal P. Fox S. E. (2011). Global profiling of rice and poplar transcriptomes highlights key conserved circadian-controlled pathways and cis-regulatory modules. *PLoS ONE* 6:e16907. 10.1371/journal.pone.0016907 21694767 PMC3111414

[B21] Goodstein D. M. Shu S. Howson R. Neupane R. Hayes R. D. Fazo J. (2012). Phytozome: a comparative platform for green plant genomics. *Nucleic Acids Res.* 40 D1178–D1186. 10.1093/nar/gkr944 22110026 PMC3245001

[B22] Greenham K. McClung C. R. (2015). Integrating circadian dynamics with physiological processes in plants. *Nat. Rev. Genet.* 16 598–610. 10.1038/nrg3976 26370901

[B23] Gross S. Martin J. Simpson J. Abraham-Juarez M. Wang Z. Visel A. (2013). De Novo transcriptome assembly of drought tolerant CAM plants, Agave deserti and Agave tequilana. *BMC Genomics* 14:563. 10.1186/1471-2164-14-563 23957668 PMC3765226

[B24] Harmer S. L. Hogenesch J. B. Straume M. Chang H. Han B. Zhu T. (2000). Orchestrated Transcription of Key Pathways in Arabidopsis by the Circadian Clock. *Science* 290 2110–2113. 10.1126/science.290.5499.2110 11118138

[B25] Hartwell J. (2005). The co-ordination of central plant metabolism by the circadian clock. *Mechanistic and Functional Studies of Proteins* 33 945–948. 10.1042/BST20050945 16246017

[B26] Hartwell J. (2006). The circadian clock in CAM plants. *Endogenous Plant Rhythms* 211–236^∗^ 10.1002/9780470988527.ch9

[B27] Hartwell J. Gill A. Nimmo G. A. Wilkins M. Jenkins J. Nimmo H. G. (1999). Phosphoenolpyruvate carboxylase kinase is a novel protein kinase regulated at the level of expression. *Plant J.* 20 333–342. 10.1046/j.1365-313X.1999.t01-1-00609.x 10571893

[B28] Higashi T. Tanigaki Y. Takayama K. Nagano A. J. Honjo M. N. Fukuda H. (2016). Detection of Diurnal Variation of Tomato Transcriptome through the Molecular Timetable Method in a Sunlight-Type Plant Factory. *Front Plant Sci* 7:87. 10.3389/fpls.2016.00087 26904059 PMC4744910

[B29] Holm S. (1979). A Simple Sequentially Rejective Multiple Test Procedure. *Scandinavian Journal of Statistics* 6 65–70.

[B30] Hotta C. T. Gardner M. J. Hubbard K. E. Baek S. J. Dalchau N. Suhita D. (2007). Modulation of environmental responses of plants by circadian clocks. *Plant Cell Environ.* 30 333–349. 10.1111/j.1365-3040.2006.01627.x 17263778

[B31] Hughes M. E. Abruzzi K. Allada R. Anafi R. C. Arpat A. Asher G. (2017). Guidelines for Genome-Scale Analysis of Biological Rhythms. *J. Biol. Rhythms* 32 380–393. 10.1177/0748730417728663 29098954 PMC5692188

[B32] Hughes M. E. Hogenesch J. B. Kornacker K. (2010). JTK_CYCLE: an efficient nonparametric algorithm for detecting rhythmic components in genome-scale data sets. *J. Biol. Rhythms* 25 372–380. 10.1177/0748730410379711 20876817 PMC3119870

[B33] Jin J. Tian F. Yang D. Meng Y. Kong L. Luo J. (2017). PlantTFDB 4.0*: toward a central hub for transcription factors and regulatory interactions in plants*. *Nucleic Acids Res* 45 D1040–D1045. 10.1093/nar/gkw982 27924042 PMC5210657

[B34] Keegan K. P. Pradhan S. Wang J. P. Allada R. (2007). Meta-analysis of Drosophila circadian microarray studies identifies a novel set of rhythmically expressed genes. *PLoS Comput Biol* 3:e208. 10.1371/journal.pcbi.0030208 17983263 PMC2098839

[B35] Kelliher C. M. Leman A. R. Sierra C. S. Haase S. B. (2016). Investigating Conservation of the Cell-Cycle-Regulated Transcriptional Program in the Fungal Pathogen. *Cryptococcus neoformans. PLoS Genet* 12 e1006453. 10.1371/journal.pgen.1006453 27918582 PMC5137879

[B36] Koda S. Onda Y. Matsui H. Takahagi K. Yamaguchi-Uehara Y. Shimizu M. (2017). Diurnal Transcriptome and Gene Network Represented through Sparse Modeling in Brachypodium distachyon. *Front Plant Sci* 8:2055. 10.3389/fpls.2017.02055 29234348 PMC5712366

[B37] Kojima S. Shingle D. L. Green C. B. (2011). Post-transcriptional control of circadian rhythms. *J. Cell Sci.* 124 311–320. 10.1242/jcs.065771 21242310 PMC3021995

[B38] Kolmos E. Chow B. Y. Pruneda-Paz J. L. Kay S. A. (2014). HsfB2b-mediated repression of PRR7 directs abiotic stress responses of the circadian clock. *Proc. Natl. Acad. Sci. U.S.A.* 111 16172–16177. 10.1073/pnas.1418483111 25352668 PMC4234549

[B39] Li G. Siddiqui H. Teng Y. Lin R. Wan X. Y. Li J. (2011). Coordinated transcriptional regulation underlying the circadian clock in Arabidopsis. *Nat. Cell Biol.* 13 616–622. 10.1038/ncb2219 21499259

[B40] Li J. Grant G. R. Hogenesch J. B. Hughes M. E. (2015). Considerations for RNA-seq analysis of circadian rhythms. *Methods Enzymol.* 551 349–367. 10.1016/bs.mie.2014.10.020 25662464

[B41] Lim C. Allada R. (2013). Emerging roles for post-transcriptional regulation in circadian clocks. *Nat. Neurosci.* 16 1544–1550. 10.1038/nn.3543 24165681

[B42] Lomb N. R. (1975). Least-squares frequency analysis of unequally spaced data. *Astrophysics and Space Science* 39 447–462. 10.1007/BF00648343

[B43] Lou P. Wu J. Cheng F. Cressman L. G. Wang X. McClung C. R. (2012). Preferential retention of circadian clock genes during diploidization following whole genome triplication in Brassica rapa. *Plant Cell* 24 2415–2426. 10.1105/tpc.112.099499 22685167 PMC3406906

[B44] Luo Z. Azencott R. Zhao Y. (2014). Modeling miRNA-mRNA interactions: fitting chemical kinetics equations to microarray data. *BMC Systems Biology* 8:19. 10.1186/1752-0509-8-19 24548346 PMC3937077

[B45] Mallona I. Egea-Cortines M. Weiss J. (2011). Conserved and divergent rhythms of crassulacean acid metabolism-related and core clock gene expression in the cactus Opuntia ficus-indica. *Plant Physiol.* 156 1978–1989. 10.1104/pp.111.179275 21677095 PMC3149932

[B46] Mateos J. L. Tilmes V. Madrigal P. Severing E. Richter R. Rijkenberg C. W. M. (2017). Divergence of regulatory networks governed by the orthologous transcription factors FLC and PEP1 in Brassicaceae species. *Proc. Natl. Acad. Sci. U.S.A.* 114 E11037–E11046. 10.1073/pnas.1618075114 29203652 PMC5754749

[B47] McClung C. R. (2013). Beyond Arabidopsis: the circadian clock in non-model plant species. *Semin. Cell Dev. Biol.* 24 430–436. 10.1016/j.semcdb.2013.02.007 23466287

[B48] Merida A. Rodriquez-Galan J. M. Vincent C. Romero J. M. (1999). Expression of the Granule-Bound Starch Synthase I (Waxy) Gene from Snapdragon Is Developmentally and Circadian Clock Regulated. *Plant Physiology* 120 401–409. 10.1104/pp.120.2.401 10364391 PMC59278

[B49] Michael T. P. Mockler T. C. Breton G. McEntee C. Byer A. Trout J. D. (2008). Network discovery pipeline elucidates conserved time-of-day-specific cis-regulatory modules. *PLoS Genet* 4:e14. 10.1371/journal.pgen.0040014 18248097 PMC2222925

[B50] Ming R. VanBuren R. Wai C. M. Tang H. Schatz M. C. Bowers J. E. (2015). The pineapple genome and the evolution of CAM photosynthesis. *Nat. Genet.* 47 1435–1442. 10.1038/ng.3435 26523774 PMC4867222

[B51] Mockler T. C. Michael T. P. Priest H. D. Shen R. Sullivan C. M. Givan S. A. (2007). The Diurnal Project: Diurnal and Circadian Expression Profiling, Model-based Pattern Matching, and Promoter Analysis. *Cold. Spring Harb. Symp. Quant. Biol* 72 353–363. 10.1101/sqb.2007.72.006 18419293

[B52] Nadimpalli S. Persikov A. V. Singh M. (2015). Pervasive variation of transcription factor orthologs contributes to regulatory network evolution. *PLoS Genet* 11:e1005011. 10.1371/journal.pgen.1005011 25748510 PMC4351887

[B53] Nimmo G. A. Nimmo H. G. Fewson C. A. Wilkins M. (1984). Diurnal changes in the properties of phosphoenolpyruvate carboxylase in Bryophyllum leaves: a possible covalent modification. *FEBS Lett.* 178 199–203. 10.1016/0014-5793(84)80600-6

[B54] Nohales M. A. Kay S. A. (2016). Molecular mechanisms at the core of the plant circadian oscillator. *Nat. Struct. Mol. Biol.* 23 1061–1069. 10.1038/nsmb.3327 27922614 PMC7750160

[B55] Nusinow D. A. Helfer A. Hamilton E. E. King J. J. Imaizumi T. Schultz T. F. (2011). The ELF4-ELF3-LUX complex links the circadian clock to diurnal control of hypocotyl growth. *Nature* 475 398–402. 10.1038/nature10182 21753751 PMC3155984

[B56] Piechulla B. (1988). Plastid and nuclear mRNA fluctuations in tomato leaves - diurnal and circadian rhythms during extended dark and light periods. *Plant Mol. Biol.* 11 345–353. 10.1007/BF00027391 24272347

[B57] Scargle J. D. (1982). Studies in astronomical time series analysis. II. Statistical aspects of spectral analysis of unevenly spaced data. *The Astrophysical Journal* 263 835–853. 10.1086/160554

[B58] Sharma A. Wai C. M. Ming R. Yu Q. (2017). Diurnal Cycling Transcription Factors of Pineapple Revealed by Genome-Wide Annotation and Global Transcriptomic Analysis. *Genome Biol. Evol.* 9 2170–2190. 10.1093/gbe/evx161 28922793 PMC5737478

[B59] Thain S. Hall A. Millar A. J. (2000). Functional independence of circadian clocks that regulate plant gene expression. *Curr. Biol.* 10 951–956. 10.1016/S0960-9822(00)00630-8 10985381

[B60] Van Bel M. Diels T. Vancaester E. Kreft L. Botzki A. Van de Peer Y. (2017). PLAZA 4.0*: an integrative resource for functional evolutionary and comparative plant genomices*. *Nucleic Acids Research* 46 D1190–D1196. 10.1093/nar/gkx1002 29069403 PMC5753339

[B61] von Caemmerer S. Griffiths H. (2009). Stomatal responses to CO2 during a diel Crassulacean acid metabolism cycle in Kalanchoe daigremontiana and Kalanchoe pinnata. *Plant Cell Environ.* 32 567–576. 10.1111/j.1365-3040.2009.01951.x 19210641

[B62] Wai C. M. VanBuren R. Zhang J. Huang L. Miao W. Edger P. P. (2017). Temporal and spatial transcriptomic and microRNA dynamics of CAM photosynthesis in pineapple. *Plant J.* 92 19–30. 10.1111/tpj.13630 28670834

[B63] Wenden B. Kozma-Bognar L. Edwards K. D. Hall A. J. Locke J. C. Millar A. J. (2011). Light inputs shape the Arabidopsis circadian system. *Plant J.* 66 480–491. 10.1111/j.1365-313X.2011.04505.x 21255161

[B64] Wilkins M. (1992). Tansley Review No. 37 Circadian rhythms: their origin and control. *New Phytologist* 121 347–375. 10.1111/j.1469-8137.1992.tb02936.x 33874151

[B65] Wyka T. P. Bohn A. Duarte H. M. Kaiser F. Luttge U. E. (2004). Perturbations of malate accumulation and the endogenous rhythms of gas exchange in the Crassulacean acid metabolism plant Kalanchoe daigremontiana: testing the tonoplast-as-oscillator model. *Planta* 219 705–713. 10.1007/s00425-004-1265-y 15127301

[B66] Yang X. Cushman J. C. Borland A. M. Edwards E. J. Wullschleger S. D. Tuskan G. A. (2015). A roadmap for research on crassulacean acid metabolism (CAM) to enhance sustainable food and bioenergy production in a hotter, drier world. *New Phytologist* 207 491–504. 10.1111/nph.13393 26153373

[B67] Yang X. Hu R. Yin H. Jenkins J. Shu S. Tang H. (2017). The Kalanchoë; genome provides insights into convergent evolution and building blocks of crassulacean acid metabolism. *Nature Communications* 8 1–15. 10.1038/s41467-017-01491-7 29196618 PMC5711932

[B68] Yeung J. Mermet J. Jouffe C. Marquis J. Charpagne A. Gachon F. (2018). Transcription factor activity rhythms and tissue-specific chromatin interactions explain circadian gene expression across organs. *Genome Res.* 28 182–191. 10.1101/gr.222430.117 29254942 PMC5793782

[B69] Yin H. Guo H. B. Weston D. J. Borland A. M. Ranjan P. Abraham P. E. (2018). Diel rewiring and positive selection of ancient plant proteins enabled evolution of CAM photosynthesis in Agave. *BMC Genomics* 19:588. 10.1186/s12864-018-4964-7 30081833 PMC6090859

[B70] Zhang R. Lahens N. Ballance H. Hughes M. E. Hogenesch J. B. (2014). A circadian gene expression atlas in mammals: Implications for biology and medicine. *PNAS* 111 16219–16224. 10.1073/pnas.1408886111 25349387 PMC4234565

