# Supplementary material for: Conservation and Diversification of Circadian Rhythmicity Between a Model Crassulacean Acid Metabolism Plant Kalanchoë fedtschenkoi and a Model C3 Photosynthesis Plant Arabidopsis thaliana
Source: Front Plant Sci. 2018 Nov 28;9:1757. doi: 10.3389/fpls.2018.01757 (PMC6279919; doi:10.3389/fpls.2018.01757)
Supplement: Supplementary file 12 [file Data_Sheet_1.docx]

Supplementary Material

Conservation and Diversification of Circadian Rhythmicity Between Crassulacean Acid Metabolism and C3 Photosynthesis Plants

Robert C. Moseley, Ritesh Mewalal, Francis Motta, Gerald A. Tuskan, Steve Haase, Xiaohan Yang^*^

*** Correspondence:** Dr. Xiaohan Yang: yangx@ornl.edu

# Supplementary Figures and Tables

## Supplementary Figures

##
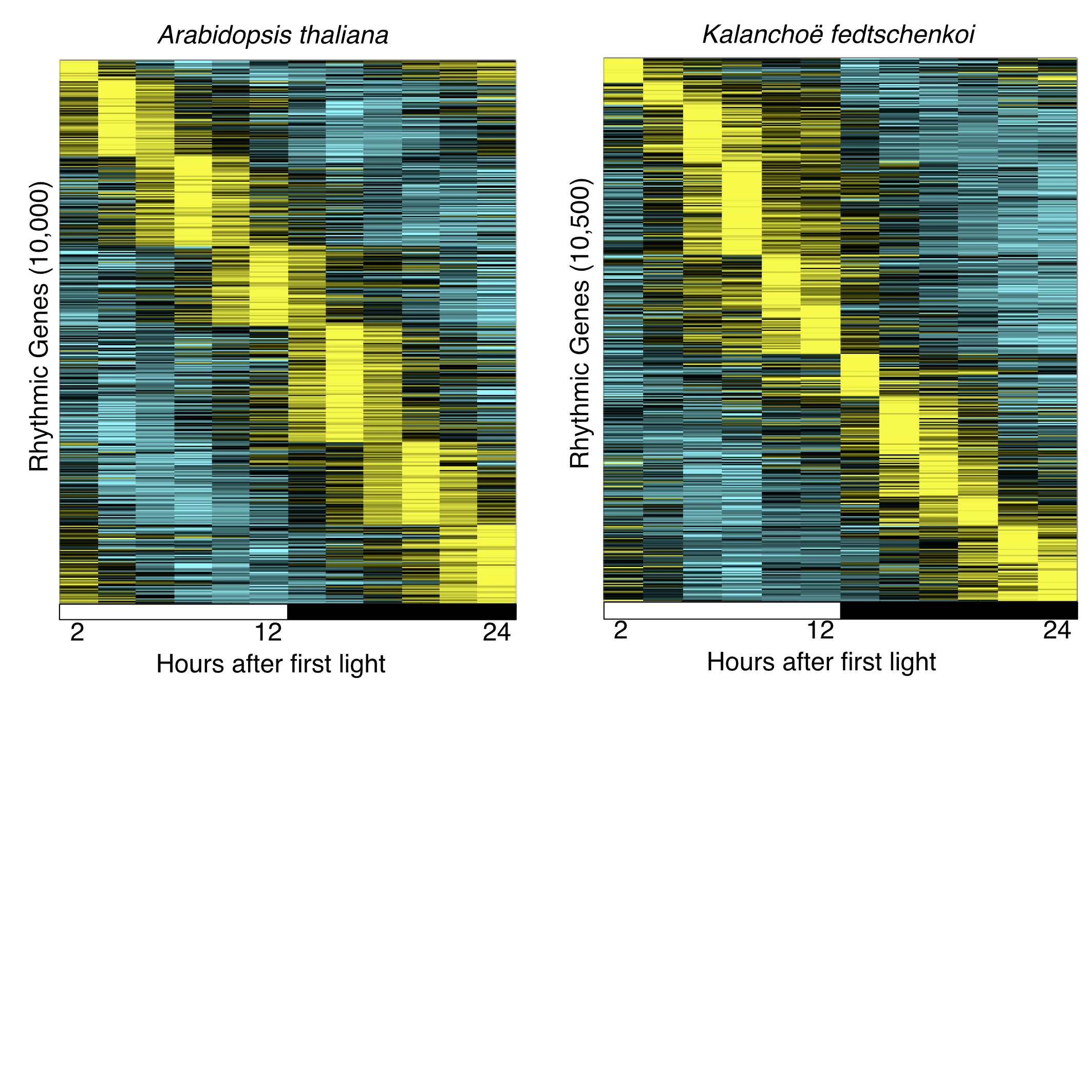


**Supplementary Figure 1. Gene identified as rhythmic through visual inspection.** Top 10,000 and 10,500 rhythmic genes in *Arabidopsis thaliana* (left) and *Kalanchoë fedtschenkoi* (right), respectively. Y-axis is genes sorted by phase. X-axis is hours after first light. Gene expression data is log transformed.


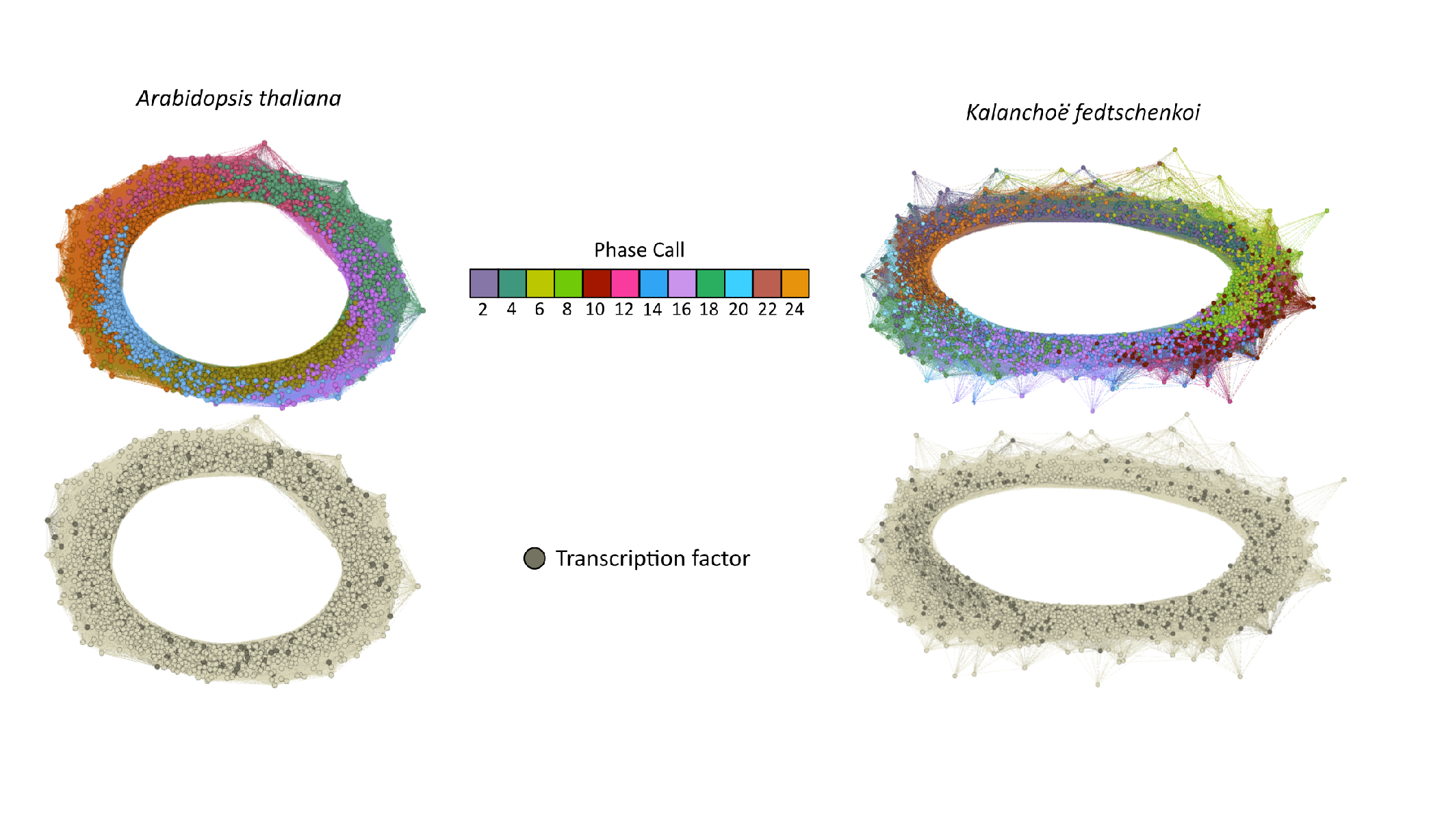


**Supplementary Figure 2. Gene co-expression networks for *Arabidopsis thaliana* and *Kalanchoë fedtschenkoi***. Networks for each species (*A. thaliana* (left) and *K. fedtschenkoi* (right)) were constructed by calculating the Spearman ranked correlation coefficient for all pair-wise combinations of gene expression in each species’ rhythmic gene set. Genes in the top multi-colored, circular networks are colored to their phase call in their diurnal expressions. Grey nodes in the bottom two-colored circular networks represent transcription factors.

## Supplementary Tables

**Supplementary Table 1. Ranking of rhythmic genes from *Arabidopsis thaliana. (****Separate file)*

**Supplementary Table 2**. **Ranking of rhythmic genes from *Kalanchoë fedtschenkoi.*** (*Separate file)*

**Supplementary Table 3**. **List of plant species used to create BLAST database.** (*Separate file)*

**Supplementary Table 4. GO enrichment of rhythmic gene sets for *Arabidopsis thaliana* and *Kalanchoë fedtschenkoi.*** *(Separate file*)

**Supplementary Table 5. Phase specific GO enrichment of rhythmic gene sets for** ***Arabidopsis thaliana* and *Kalanchoë fedtschenkoi.*** *(Separate file*)

**Supplementary Table 6. Placement of *Arabidopsis thaliana* and *Kalanchoë fedtschenkoi genes in ortholog and gene Types*. *(****Separate file)*

**Supplementary Table 7. GO enrichment of *Arabidopsis thaliana* and *Kalanchoë fedtschenkoi genes in each ortholog group type.* *(****Separate file)*

**Supplementary Table 8. Supplementary Table 8. Carboxylation genes in *Kalanchoë fedtschenkoi* and their orthologs in *Arabidopsis thaliana*. *(****Separate file)*

**Supplementary Table 9. Supplementary Table 9. Decarboxylation genes in *Kalanchoë fedtschenkoi* and their orthologs in *Arabidopsis thaliana*. *(****Separate file)*

**Supplementary Table 10. *Kalanchoë fedtschenkoi* genes that were either not placed in an orthogroup or not placed in an orthogroup with an *Arabidopsis thaliana* gene had their protein sequence BLAST against various plant proteomes.** *(Separate file)*

**Supplementary Table 11. *Kalanchoë fedtschenkoi-* and CAM-specific genes in type 5 ortholog group.** *(Separate file)*

**Supplementary Table 12. *Kalanchoë fedtschenkoi* orthologs of *Arabidopsis thaliana* core clock genes.**

| *A. thaliana* Genes | *A. thaliana* Gene Symbols | *K. fedtschenkoi* Genes |
| --- | --- | --- |
| AT3G54500 | LNK2 | Kaladp0060s0264 |
| AT3G54500 | LNK2 | Kaladp0099s0129 |
| AT5G52660 | RVE6 | Kaladp0019s0045 |
| AT5G52660 | RVE6 | Kaladp0022s0168 |
| AT5G52660 | RVE6 | Kaladp0055s0349 |
| AT5G64170 | LNK1 | Kaladp0047s0123 |
| AT5G64170 | LNK1 | Kaladp0607s0046 |
| AT2G46790 | PRR9 | Kaladp0032s0115 |
| AT5G08330 | CHE | Kaladp0032s0054 |
| AT1G22770 | GI | Kaladp0040s0489 |
| AT5G24470 | PRR5 | Kaladp0032s0115 |
| AT5G02810 | PRR7 | Kaladp0101s0041 |
| AT2G40080 | ELF4 | Kaladp0037s0163 |
| AT2G40080 | ELF4 | Kaladp0045s0206 |
| AT2G40080 | ELF4 | Kaladp0059s0037 |
| AT3G46640 | LUX | Kaladp0033s0047 |
| AT5G61380 | TOC1 | Kaladp0040s0446 |
| AT2G25930 | ELF3 | Kaladp0039s0732 |
| AT2G46830 | CCA1 | Kaladp0066s0115 |
| AT2G46830 | CCA1 | Kaladp0496s0018 |
| AT1G01060 | LHY | Kaladp0066s0115 |
| AT1G01060 | LHY | Kaladp0496s0018 |
| AT3G09600 | RVE8 | Kaladp0577s0020 |
| AT1G12910 | LWD1 | Kaladp0048s0797 |
| AT3G26640 | LWD2 | Kaladp0048s0797 |
| AT5G59570 | NOX | Kaladp0033s0047 |
